# Paramutation-Like Interaction of T-DNA Loci in Arabidopsis

**DOI:** 10.1371/journal.pone.0051651

**Published:** 2012-12-14

**Authors:** Weiya Xue, Colin Ruprecht, Nathaniel Street, Kian Hematy, Christine Chang, Wolf B. Frommer, Staffan Persson, Totte Niittylä

**Affiliations:** 1 Department of Forest Genetics and Plant Physiology, Umeå Plant Science Centre, Swedish University of Agricultural Sciences, Umeå, Sweden; 2 Max-Planck-Institute of Molecular Plant Physiology, Potsdam, Germany; 3 Department of Plant Physiology, Umeå Plant Science Centre, Umeå University, Umeå, Sweden; 4 Institut Jean-Pierre Bourgin, INRA-AgroParisTech, Versailles, France; 5 Department of Plant Biology, Carnegie Institution for Science, Stanford, California, United States of America; University of Michigan, United States of America

## Abstract

In paramutation, epigenetic information is transferred from one allele to another to create a gene expression state which is stably inherited over generations. Typically, paramutation describes a phenomenon where one allele of a gene down-regulates the expression of another allele. Paramutation has been described in several eukaryotes and is best understood in plants. Here we describe an unexpected paramutation-like *trans* SALK T-DNA interaction in Arabidopsis. Unlike most of the previously described paramutations, which led to gene silencing, the *trans* SALK T-DNA interaction caused an increase in the transcript levels of the endogenous gene (*COBRA*) where the T-DNA was inserted. This increased *COBRA* expression state was stably inherited for several generations and led to the partial suppression of the *cobra* phenotype. DNA methylation was implicated in this *trans* SALK T-DNA interaction since mutation of the DNA methyltransferase 1 in the suppressed *cobra* caused a reversal of the suppression. In addition, null mutants of the DNA demethylase *ROS1* caused a similar *COBRA* transcript increase in the *cobra* SALK T-DNA mutant as the *trans* T-DNA interaction. Our results provide a new example of a paramutation-like *trans* T-DNA interaction in Arabidopsis, and establish a convenient hypocotyl elongation assay to study this phenomenon. The results also alert to the possibility of unexpected endogenous transcript increase when two T-DNAs are combined in the same genetic background.

## Introduction

Epigenetic modifications can be defined as heritable information that is not encoded in the nucleotide sequence of DNA. An important epigenetic mark is cytosine methylation of DNA, as severe defects in DNA methylation in mammals are embryonic lethal and in plants lead to pleiotropic morphological defects [1]. To avoid these deleterious effects, DNA methylation patterns are carefully maintained and stably inherited.

DNA methylation has also been implicated in paramutation [2]–[4] where specific DNA sequences interact in *trans* to establish meiotically heritable gene expression states [5]. The maize *b1* locus encoding a transcription factor regulating anthocyanin biosynthesis provides a classic example of paramutation. Two alleles of the *b1* locus, the *B′* and *B-I*, are involved in paramutation. The *B-I* allele has a high and the *B′* low level of expression and when *B-I* and *B′* are combined in the same nucleus the *B-I* gets converted to *B′* [6], [7], [8]. A hepta-repeat DNA sequence required for the *B-I* to *B′* paramutation is located approximately 100 kb upstream of the transcription start site of *b1* [9]. Several other paramutation loci have been documented in maize and other plants (reviewed in [5]). Paramutation has also been described at the tyrosine kinase receptor encoding *Kit* locus in mice [10] indicating that the phenomena occurs across eukaryotes. The exact mechanism of paramutation is not clear but has been shown to involve RNA mediated transfer of information between paramutagenic and paramutable alleles in both plants and animals [10], [11], [12], [13]. This has led to models where RNA directed DNA methylation (RdDM) is responsible for the differential DNA methylation observed in paramutation [6], [14].

In plants, cytosine DNA methylation is found in all sequence contexts (CG, CHG and CHH) and several enzymes involved in DNA methylation have been identified. Existing DNA methylation is maintained by three different pathways: DNA METHYLTRANFERASE 1 (MET1) maintains CG methylation, CHROMOMETHYLASE 3 (CMT3) maintains CHG methylation [15] and CHH methylation is maintained by DOMAINS REARRANGED METHYLTRANSFERASE 1 and 2 (DRM1 and DRM2) [16], [17]. *De novo* methylation of previously unmethylated sequences is also carried out by DRM2 [16]. Plants also have a mechanism to remove DNA methylation, for example through the REPRESSOR OF SILENCING1 (ROS1) DNA demethylase activity [18]. The final DNA methylation pattern of a genome is established by the combined activity of DNA methyltransferases and demethylases [19].

We discovered that non-allelic SALK T-DNA insertions in Arabidopsis genome can interact in *trans* and cause epigenetic changes creating a DNA methylation dependent paramutagenic allele in the process. DNA methylation mediated *trans* T-DNA interactions, where one T-DNA induces an epigenetic silencing effect on a second T-DNA, have previously been documented in tobacco [20]. A similar *trans* silencing T-DNA effect was also observed in Arabidopsis and attributed to the presence of the cauliflower mosaic virus 35S promoter in the SALK T-DNA inserts [21]. In our case the SALK T-DNA triggered epigenetic changes led to increased expression of the endogenous locus where the T-DNA was residing, and in the process this locus became paramutagenic. Characterisation of this SALK T-DNA interaction indicated the involvement of DNA methylation, which was modulated by MET1 and possibly ROS1. The results alert to an unexpected phenomenon associated with T-DNA insertions and describe a new paramutagenic interaction in Arabidopsis.

## Results

### Suppression of the Primary Cell Wall *cobra* T-DNA Insertion Mutant

**Figure 1:**
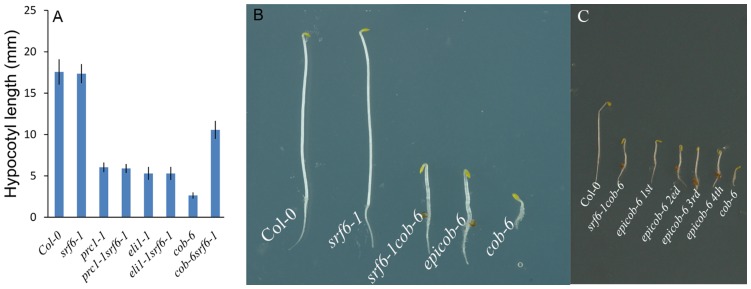
*srf6* SALK T-DNA triggered suppression of *cobra* phenotype and inheritance of *epicob-6*. (**A**) Quantification of hypocotyl length in four-day-old dark grown seedlings. Genotypes, mean and SE are indicated, n = 30–40. (**B**) Four-day-old dark grown seedlings of Col-0, *srf6-1*, *srf6-1cob-6*, *epicob-6* and *cob-6*. (**C**) Phenotype comparison of etiolated *epicob-6* for four generations.

The concept that co-expressed genes tend to be functionally related [22] led us to investigate the genetic interactions in the primary cell wall co-expressed gene network of Arabidopsis (Figure S1) [23]. We discovered that SALK T-DNA mutants of the receptor-like kinase *SRF6* (*srf6-1* and *srf6-3*) were able to partially suppress the growth defect of the cellulose deficient mutant *cobra* (*cob-6*) [24], but did not suppress mutants of *CELLULOSE SYNTHASE 6* (*prc1)* [25] or *CELLULOSE SYNTHASE 3* (*eli1)* [26] (Figure 1A and Figure S2). *cob-6* carries a SALK T-DNA insertion in the first intron of the *COBRA* gene [24] whereas *prc1* and *eli1* contain a single nucleotide change in the corresponding gene [25], [26]. The locus of the different *srf6* and *cob* alleles used in this study are illustrated in Figure S3. COBRA is an extracellular glycosylphosphatidyl inositol anchored protein, which is essential for cellulose synthesis and anisotropic growth [27]. The *cob* phenotype is consequently most obvious in young roots and dark grown hypocotyls (Figure 1B) [28]. Etiolated *srf6-1cob-6* double-mutant seedlings contained higher levels of cellulose compared to *cob-6* mutants (Figure S4) establishing that the suppression mechanism was partially complementing the cellulose biosynthesis defect in *cob-6*. *srf6* null mutants did not show any visible growth phenotypes on their own (Figure 1B and Figure S2).

### Epigenetic Inheritance of *cob-6* Suppression

The segregation ratio of the F2 progeny from the cross between *srf6-1* and *cob-6* deviated substantially from the expected for recessive mutations, which would be one suppressed *cob-6* seedling per 16 seedlings. Instead we observed one suppressed *cob-6* seedling per ca. four seedlings (N = 228 of which 60 were suppressed homozygous *cob-6*) in the F2 progeny. The same result was also obtained with a second *SRF6* knock-out allele *srf6-3.* To clarify the mechanism of this unusual phenotypic segregation ratio, and the genetic interaction between *SRF6* and *COBRA*, we genotyped the F2 plants. We discovered that the *cob-6* suppressor phenotypes were always homozygous for *cob-6* but either wild-type, hetero- or homozygous for *srf6*. Hence, *cob-6* single and *srf6cob-6* double mutants showed a very similar suppressed phenotype in the F2 progeny of the *srf6*×*cob-6* cross. To further investigate the suppressor mechanism we backcrossed the *srf6-1cob-6* double mutant with the parental *cob-6* and surprisingly found that the F1 plants still showed the suppressed *cob-6* phenotype (Figure S5). Thus, once the *cob-6* suppression was established even a wild-type copy of *SRF6* was unable to completely reverse the suppression. These results, together with the deviant F2 segregation from the *srf6*×*cob-6* cross, suggested that *srf6* acts dominantly and suppresses the *cob-6* phenotype through an epigenetic mechanism.

To distinguish between the original *cob-6* line and the suppressed *cob-6* lines with wild-type *SRF6* locus derived from the F2 of the *srf6*×*cob-6* cross, the suppressed *cob-6* lines were named *epicob-6* (Figure 1B and Table S1). The etiolated *epicob-6* hypocotyls were slightly shorter than the *srf6cob-6* double mutant suggesting that the *srf6* allele had a small additional effect on the phenotype. The *epicob-6* plants were grown for four generations but no reversion back to *cob-6* phenotype was observed (Figure 1C and Table S2). Hence, *epicob-6* can be inherited to progeny independent of the *srf6* mutation, and this inheritance is stable for at least four generations.

### Increased *COBRA* Transcript Level Explained *cob-6* Suppression

**Figure 2:**
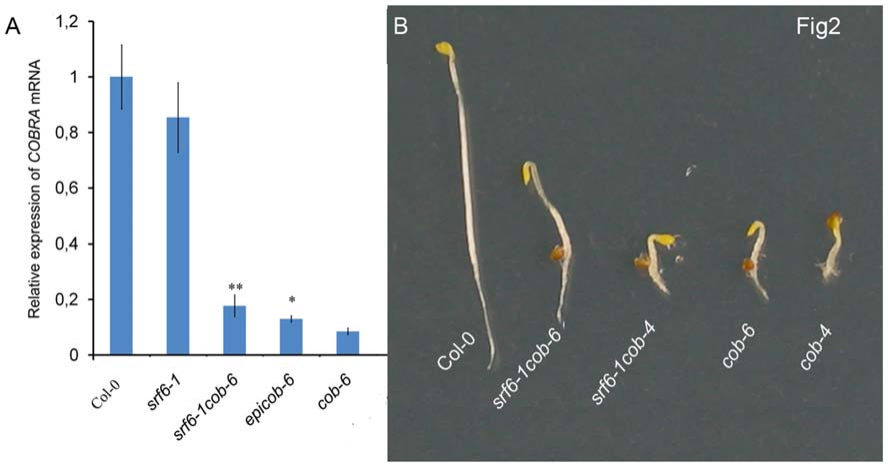
*COBRA* transcript level increase in the suppressed *cobra*. (**A**) qPCR determined *COBRA* transcript level in etiolated seedlings grown on ½ MS medium. Genotypes, mean and SE are indicated, n = 3 pools of seedlings. Asterisks indicate *P* values for comparison with *cob-6*: * *P*<0.05; ** *P*<0.001 (Student’s *t*-test). (**B**) Phenotype comparison of etiolated *srf6-1cob-6* and *srf6-1cob-4* seedlings. Pictures are representative of multiple plants for each genotype.

A *cob* null mutant is seedling lethal [28] but homozygous *cob-6* plants are viable, and produce viable seeds [24]. The *cob-6* phenotype was fully complemented by a genomic fragment of the *COBRA* gene (Figure S6), confirming that the *cob-6* phenotype is due to the T-DNA insert in the first intron of the *COBRA* gene. We tested for the possibility that the T-DNA-containing intron could be correctly spliced out in *cob-6*. Indeed, we were able to amplify the full-length cDNA from *cob-6* plants (Figure S7). Sequencing of this *COBRA* cDNA showed that it encodes for a wild-type COBRA protein (data not shown). Quantitative real-time PCR (qPCR) experiments using primers amplifying across the intron containing the *cob-6* T-DNA showed that *COBRA* mRNA levels in *cob-6* mutant are about 10% of wild type (Figure 2A). Since the *cob-6* mutant only resulted in reduced *COBRA* mRNA, it is plausible that the suppressed *cob-6* phenotype could be due to a change in *COBRA* mRNA levels, especially since most of the described epigenetic phenomena affect gene expression [29], [30]. We, therefore, compared *COBRA* mRNA levels in Col-0, *srf6-1*, *cob-6*, *srf6-1cob-6*, and *epicob-6* (Figure 2A). *srf6-1cob-6* and *epicob-6* displayed a significant increase of *COBRA* mRNA compared to *cob-6*, approx. 20% and 17% of wild-type *COBRA* mRNA levels, respectively. To confirm that the suppressed *cobra* phenotype was due to increased levels of *COBRA* transcript we crossed *srf6-1* with a complete knock-out of *COBRA* (*cob-4*) [28]. The *cob-4* mutant was not suppressed by *srf6-1* (Figure 2B). These results established that *srf6* SALK T-DNA mutations suppress the *cob-6* SALK T-DNA knock-down mutant through a transcript increase mechanism.

### Epigenetic *cob-6* Suppression is Caused by *trans* SALK T-DNA Interaction

We suspected that the *cob-6* suppression was linked to the SALK T-DNA insertion in the *srf6* lines rather than the *SRF6* defect. To test this we crossed *cob-6* with three randomly selected SALK T-DNA insertion lines and a SAIL T-DNA insertion in the *SRF6* homologue *SRF4.* All three SALK T-DNA lines suppressed *cob-6* to varying degrees, but the *srf4-1* SAIL line had no effect on the *cob-6* phenotype (Table 1). Hence it appeared that the SALK T-DNAs could somehow interact with each other to promote *COBRA* expression. To further test this hypothesis we obtained a premature stop codon allele of *SRF6* (*srf6-4*) from a Landsberg TILLING population (Figure S3) and crossed this line to a *cob-6,* which had been backcrossed to Landsberg five times. No suppressed *cob-6* plants were observed in the F2 progeny of the cross between the *cob-6* in Landsberg and *srf6-4* (Figure S8). Thus it could be concluded that the *cob-6* suppression is caused by a dominant *trans* interaction of SALK T-DNA insertions.

**Table 1:** Hypocotyl length of the randomly selected SALK T-DNA *r1, r2* and *r3cob-6* and the *srf4-1* SAIL T-DNA *cob-6* double mutants.

Genotype	Genomic locus of the additional T-DNA	Number of plants	Three-day-old etiolated hypocotyl length (mm)*
*cob-6*	–	29	1.5±0.1Aa
*srf4-1cob-6*	AT3G13065	30	1.6±0.1Aa
*r1cob-6*	AT3G30980	25	3.4±0.2Bb
*r2cob-6*	AT1G56340	21	2.4±0.1Cc
*r3cob-6*	AT2G35050	29	3.3±0.1Bb

* A, B and C indicate ranking by Duncan test at *P*≤0.01;

a, b and c indicate ranking by Duncan test at *P*≤0.05.

**Figure 3:**
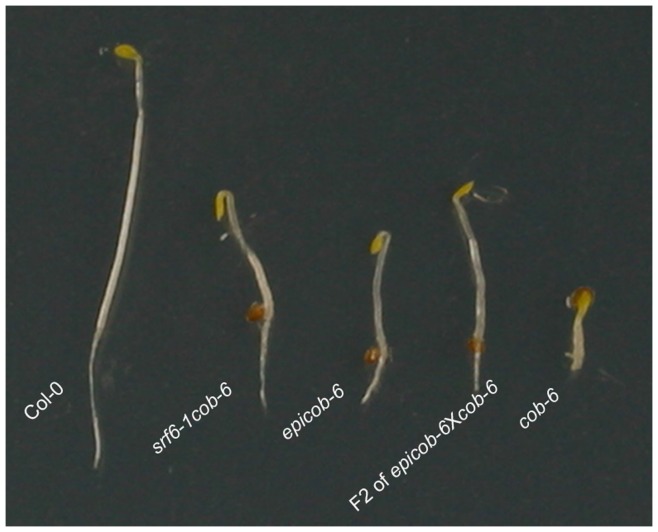
Transmission of *epicob-6* phenotype. Four-day-old etiolated seedlings in the F2 progeny from a cross between *epicob-6* and *cob-6.* Genotypes are indicated. Pictures are representative of multiple plants for each genotype.

To further elucidate the SALK T-DNA mediated epigenetic effects, we crossed *epicob-6* to *cob-6* and Col-0. In the F1 and F2 population derived from the cross between *epicob-6* and *cob-6*, all the plants showed suppression of the *cob-6* phenotype (Figure 3A and Table S3). This result showed that the *epicob-6* established by the *srf6* SALK T-DNA was able to convert *cob-6* into the suppressed *epicob-6* state. Therefore the *epicob-6* suppressor state behaved similarly to a paramutagenic allele in that it could convert a *cob-6* paramutable allele to a higher expression state. Interestingly, in the F2 population of *epicob-6*×Col-0 the suppression was lost and only Col-0 and *cob-6* were observed, suggesting that two allelic copies of the *cob-6* T-DNA were required for the maintenance of the suppression state (Table S3).

### Increased *COBRA* Expression in *cob-6* was Associated with Increased DNA Methylation

**Figure 4:**
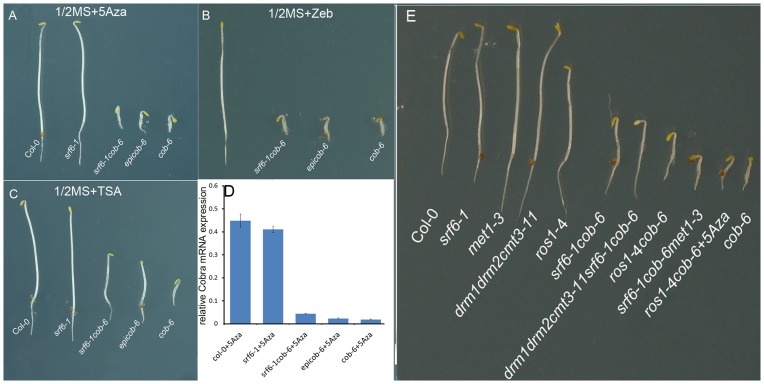
The effect of epigenome modification on *srf6* SALK T-DNA caused *cob-6* suppression. Etiolated seedlings grown on ½ MS medium with (**A**) 30 µM of DNA methylation inhibitor 5-Azacytidine (5Aza) (**B**) 30 µM of DNA methylation inhibitor zebularine (Zeb). (**C**) 1.6 µM of histone deacetylase inhibitor Trichostatin A (TSA). Genotypes are indicated. Pictures are representative of multiple plants for each genotype. (**D**) qPCR determined *COBRA* transcript level in etiolated seedlings grown on ½ MS medium with 30 µM 5-azacytidine (5-Aza) normalised against Col-0 without 5-Aza. Genotypes, mean and SE are indicated, n = 3 pools of seedlings. (**E**) The effect of DNA methylation mutants on *srf6* SALK T-DNA caused *cob-6* suppression. Genotypes are indicated. Pictures are representative of multiple plants for each genotype.

To assess the epigenetic nature of the SALK T-DNA mediated increase in *COBRA* transcript levels in *cob-6*, we analysed whether DNA methylation or histone acetylation could be involved in this process. We grew etiolated seedlings on solid growth media containing the DNA methylation inhibitors 5-azacytidine and zebularine, and the histone deacetylase inhibitor trichostatin A (TSA). 5-azacytidine and zebularine reduce DNA methylation levels through deactivating DNA methyltransferases [31], [32], and TSA leads to increased acetylation of histones [33]. We discovered that the *srf6-1cob-6* and *epicob-6* mutant reversed to *cob-6* phenotype when grown on either 5-azacytidine (Figure 4A), or zebularine (Figure 4B), but not on TSA (Figure 4C). The 30 µM of 5-azacytidine or zebularine had no visible effects on the wild-type phenotype (Figure 4A and 4B). We also measured the *COBRA* mRNA levels in the *srf6-1cob-6* seedlings from the 5-azacytidine experiment and established that 5-azacytidine can repress the *COBRA* expression in *srf6-1cob-6* and *epicob-6* seedlings (Figure 4D).

Several proteins have been identified that affect DNA methylation in Arabidopsis. The DNA methyltransferases DRM1, DRM2 and CMT3 are involved in *de novo* DNA methylation and in CHG methylation, respectively [15], [16]. MET1 is a methyl transferase thought to be primarily responsible for maintenance of CG methylation [34]. We crossed the *srf6-1cob-6* with *met1-3* and *drm1drm2cmt3-11* mutants. Interestingly, we found *cob-6* mutant phenotypes in seedlings from the F2 progeny of the cross between *srf6-1cob-6* and *met1-3*. The genotypes of the plants displaying the *cob-6* phenotypes were either homo- or heterozygous for *met1-3* and *srf6-1* and homozygous for *cob-6* (Figure 4E and Table 2). It is not unexpected that the heterozygous *met1-3* can reverse the *cob-6* suppression since the heterozygous *met1-3* has been shown to cause DNA methylation changes [35]. Also the *srf6-1cob-6drm1-1drm2-2cmt3-11* mutant seedlings showed a small but significant reversal in the suppression of the *cob-6* phenotype (Figure 4E and Table 2). These data indicated that an increase in DNA methylation was involved in the SALK T-DNA interaction triggered suppression of the *cob-6* phenotype, and that this methylation mark was removed in the *met1-3* background and in seedlings treated with methylation inhibitors and reduced in the *drm1-1drm2-2cmt3-11* background.

**Table 2:** Effect of the different DNA methylation mutants on the *trans cob-6* SALK T-DNA interaction.

Genotype		Number of plants	Four-day-old etiolated hypocotyl length (mm)*
Col-0	35		11.0±0.2Aa
*ros1-4*	47		11.0±0.2Aa
*drm1drm2cmt3*	41		11.0±0.2Aa
*srf6-1cob-6*	46		6.2±0.2Bb
*drm1drm2cmt3-11srf6-1cob-6*	32		4.1±0.1Cc
*ros1-4cob-6*	46		4.6±0.1Dd
*met1-3srf6-1cob-6*	9		1.7±0.1Ee
*cob-6*	50		1.8±0.1Ee

* A, B,C,D,E indicate ranking by Duncan test at *P*≤0.01;

a,b,c,d,e indicate ranking by Duncan test at *P*≤0.05.

### Mutation in the DNA Demethylase ROS1 also Suppressed *cob-6*

We tested whether the increased DNA methylation responsible for *cob-6* suppression could be established through a decreased DNA demethylation activity. ROS1 is a DNA demethylase that prevents DNA hypermethylation of both endogenous genes and transgenes [18], [36], [37]. We crossed *cob-6* with a SAIL T-DNA insertion line in *ROS1* (*ros1-4*) and discovered that the phenotype of *ros1-4cob-6* was also suppressed, similar to *srf6cob-6,* and that the phenotype also responded to 5-azacytidine (Figure 4E). Furthermore, the transcript level of *COB1* was elevated in *ros1-4cob-6* (Figure S9). However, unlike in the progeny of *srf6* x *cob-6,* the *ros1-4* mutation suppressed *cob-6* only in the homozygous *ros1-4cob-6* lines and did not create the *epicob-6* phenotype. A possible reason for the lack of *epicob-6* in the *ros1-4* cross is that the SALK T-DNAs act dominantly in establishing *cob-6* suppression, whereas the *ros1-4* SAIL T-DNA and other *ros1* mutations are recessive [18]. Hence the heterozygous *ros1-4* is not able to create the paramutagenic *epicob-6* allele. We observed no significant additive *cob-6* suppression effect in a *srf6-1ros1-3cob-6* triple mutant compared to *srf6-1cob-6* suggesting that the SALK *trans* T-DNA suppressor effect acts on the same locus as ROS1 (Table S4).

## Discussion

We discovered a *trans* interaction of SALK T-DNA insertions in Arabidopsis, which led to increased transcript levels of the endogenous gene in the SALK T-DNA insertion site. The affected SALK T-DNA allele (*cob-6*) was an intron insertion in *COBRA* gene, which is required for cellulose biosynthesis [28]. While preparing this manuscript Gao and Zhao published a very similar observation but with a different set of SALK T-DNA insertion mutants indicating that such T-DNA interactions are not unusual and may represent a common phenomenon [38]. Furthermore Gao and Zhao also observed suppression of the *cob-6* root phenotype by a SALK T-DNA insertion in the auxin biosynthesis gene *YUCCA1* [38]. In both our study and Gao and Zhao (2012) the transcript level increase of the endogenous gene occurred in lines where the SALK T-DNA was inserted in an intron. In both cases this intron insertion caused a reduction in the transcript levels of the endogenous gene, which was partially rescued by the *trans* SALK T-DNA interaction. A SAIL T-DNA insertion in *SRF4* did not induce *cob-6* suppression (Table 1) suggesting that the *trans* T-DNA interaction may require the sequence similarity between two SALK T-DNAs.

Inhibitors of DNA methylation were able to reverse the *trans* SALK T-DNA interaction induced transcript increase suggesting that DNA methylation was responsible for the increased expression of the endogenous locus (Figure 4). The involvement of DNA methylation was confirmed by introducing a mutation in the main maintenance DNA methyl transferase MET1 into the *srf6cob-6*. The *srf6-1cob-6met1* mutant seedlings were phenotypically identical to the original *cob-6* and *srf6cob-6,* as were the *epicob-6* treated with DNA methylation inhibitors. MET1 is therefore involved in the maintenance of the *trans* SALK T-DNA interaction induced *cob-6* suppression. A SAIL T-DNA mutation of the *ROS1* DNA demethylase had a similar effect on *cob-6* phenotype and *COB* expression as the *trans* SALK T-DNA interaction (Figure 4E, Figure S9 and Table S4). The *COB* expression was not changed in *ros1-4,* and the *ros1-4* mutant had no additive effect on the suppression of *cob-6* implying that both ROS1 and the *trans* SALK T-DNA effect acted on the same locus. Based on the observation that several SALK T-DNAs were able to trigger the suppression of the *cob-6* while an EMS induced *srf6* null mutant was not (Table 1 and Figure S8), we hypothesise that the *cob-6* T-DNA is most likely the target of the suppressor modifications. Consequently our data also suggested that already the *cob-6* T-DNA alone has a tendency to become methylated but that this is counteracted by ROS1 activity.

Once established the *cob-6* suppressor locus became paramutagenic in that the *epicob-6* was able to convert *cob-6* to *epicob-6* (Figure 3 and Table S3). The *COB* transcript level was significantly increased in *epicob-6* compared to *cob-6* (Figure 2A). We suspected that the increase in *COB* transcript levels is due to a secondary effect of the *trans* SALK T-DNA interaction. The 35S promoter in *cob-6* T-DNA may result in the expression of a *COB* antisense transcript, which is reduced in response to the paramutation causing an increase in the wild-type *COB* transcript. The fact that the SAIL T-DNA lines, *srf4-1* or heterozygous *ros1-4,* which do not contain a 35S promoter were unable to suppress *cob-6* suggested the 35S promoter homology or activity may be causing the suppression and the paramutation effect. In support of this hypothesis the cauliflower mosaic virus 35S promoter in the SALK T-DNA inserts has also previously been linked to *trans* T-DNA effects in Arabidopsis [21]. Another possible factor influencing the degree of the suppression might be the locus of the T-DNA insertion. It is important to note that the randomly selected additional SALK T-DNA loci displayed a relatively minor suppression of the *cob-6* phenotype compared to the two *srf6* SALK-alleles (Table 1 and Table 2). Thus, several components might affect the proposed *trans* SALK T-DNA interaction mechanism.

Interestingly, when *epicob-6* was crossed to Col-0 the phenotype of the *epicob-6* reverted back to the original *cob-6* phenotype indicating that homozygosity of the *cob-6* T-DNA allele was important for the maintenance of the paramutagenic *epicob-6* allele (Table S3). This suggested that the maintenance of the paramutagenic *epicob-6* may require the presence of a second paramutagenic (*epicob-6*) or paramutable (*cob-6*) allele to be introduced during fertilisation. The fact that mutation of the ROS1 demethylase could also suppress the *cob-6* phenotype (Figure 4E and Table S4) implied that the *cob-6* T-DNA is actively demethylated. It is therefore possible that ROS1 is involved in reverting the *epicob-6* back to *cob-6* after the cross of *epicob-6* to Col-0. The mechanism of this allele effect and involvement of ROS1 in the *cob-6* SALK T-DNA suppression deserve further study. Our results establish a new Arabidopsis system where this question and the *trans* SALK T-DNA paramutation phenomena can be studied with a convenient hypocotyl elongation assay as a reporter.

## Materials and Methods

All Arabidopsis lines used in this study are in accession Columbia with the exception of *srf6-4,* which is in accession Landsberg *glabra*. For the cross with *srf6-4* the *cob-6* mutation was introduced to the Landsberg background by backcrossing the *cob-6* to Landsberg *erecta* five times. All Arabidopsis lines are listed in Table S5. Plants were grown in a growth chamber with 16 hours light (150 µmolm^−2^s^−1^) and 8 hours dark, temperature 22°C (day) and 18°C (night), relative humidity 60–70%. Etiolated seedlings were first stratified for 2 days at 4°C and then grown on ½ MS medium for 3–5 days in the dark. For inhibitor experiments the respective inhibitor was added directly to the MS medium using the indicated final concentrations. All the primers used in this study are listed in Table S5.

### Complementation of *cob-6*

A fragment containing 1.3 kb upstream the transcription start site of *COBRA* and the whole *COBRA* gene was isolated by PCR and cloned into the binary vector pGWB1 [39]. The construct was introduced into the Agrobacterium strain GV3101 and transformed into *cob-6* plants. Several T2 homozygote lines were grown for phenotyping.

### Analysis of *COBRA* Expression

RNA was isolated from three to nine 20 mg (fresh weight) pools of etiolated seedlings using the QIAGEN RNeasy mini kit (QIAGEN, www.qiagen.com). All expression experiments were repeated a minimum of three times with similar results. About 500 ng total RNA was reverse-transcribed in 20 µl reaction volume using the Bio-Rad iScript cDNA Synthesis Kit (Bio-Rad, www.bio-rad.com). The quantitative RT-PCR was performed with 0.1 µl cDNA 0.25 µM gene specific primers and 10 µl SYBR Green Master Mix (Bio-Rad iQ SYBR Green Supermix) in 20 µl reaction volume on Roche Lightcycler 480. The quantification was done according to the advanced relative quantification method [40], and the *HELICASE* reference gene (AT1G58050) chosen after an evaluation of reference genes according to [41] and [42]. The primers used for *COBRA* expression spanned the *cob-6* T-DNA inserted in the first intron.

### Cellulose Measurement

To determine the crystalline cellulose content, etiolated seedlings were transferred to 2 ml reaction tubes and treated with Updegraff-reagent [43]. On the resulting pellet Seaman hydrolysis [43] was performed and the hexose content was determined with the anthrone assay described in [44].

## Supporting Information

Figure S1 **Truncated co-expression network from Cluster 86 in** [**23**] **.** Brief annotations of genes are indicated in black text. Different coloured edges indicate strength of transcriptional coordination. Green; mutual rank ≤10, Orange; mutual rank ≤20, Red; mutual rank ≤30. Low mutual rank indicates stronger co-expression relationships. Coloured nodes indicate embryo lethality (red), other described phenotypes (green), and no reported phenotype (grey) of mutants corresponding to the respective gene.
(TIF)

Figure S2 **Phenotype of six-week-old Col-0,** ***srf6-1, cob-6*** **and** ***srf6-1cob-6*** **plants grown in 16-h light, 8-h dark.** Scale bar 5 cm.
(TIF)

Figure S3 **Location of the premature stop codon in** ***srf6-4*** **and the T-DNA insertions in** ***COBRA*** **and** ***SRF6*** **.**
(TIFF)

Figure S4 **Cellulose content in four-day-old dark grown seedlings.** Genotypes, mean and SE are indicated. A, B, and C indicate significant difference of the genotypes ranked by Duncan’s test at P<0.01, a, b, c indicate ranking by Duncan test at *P*≤0.05.
(TIFF)

Figure S5 **The phenotype of etiolated F1 seedlings derived from the cross between** ***srf6-1cob-6*** **and** ***cob-6*** **.** Picture is representative of multiple seedlings.
(TIF)

Figure S6 **Complementation of** ***cob-6*** **with a genomic** ***COBRA*** **construct.**
(TIF)

Figure S7 **Amplification of full length** ***COBRA*** **cDNA from** ***cob-6*** **and Col-0.**
(TIF)

Figure S8 **Phenotype comparison of the** ***srf6-4cob-6*** **and** ***cob-6*** **etiolated seedlings.** Picture is representative of multiple seedlings.
(TIF)

Figure S9 **The effect of DNA demethylase** ***ros1-4*** **mutation on** ***COBRA*** **transcript levels.** Also shown is the effect of DNA methylation inhibitor 5-azacytidine (5-Aza) on *COBRA* expression in *ros1-4* and *ros1-4cob-6.* Genotypes, mean and SE are indicated. RNA was extracted from etiolated seedlings, n = 3 pools of seedlings.
(TIF)

Table S1 **Hypocotyl length of Col-0,** ***srf6-1, srf6-1cob-6, epicob-6*** **and** ***cob-6.***
(PPTX)

Table S2 **Hypocotyl length of the different** ***epicob-6*** **generations.**
(PPTX)

Table S3 **Hypocotyl length of the** ***epicob-6*** **and the F2 plants derived from** ***epicob-6*** **crossed with** ***cob-6.*** And the *cob-6* and F2 *cob-6* plants derived from *epicob-6* crossed with Col-0
(PPTX)

Table S4 **Hypocotyl length of three-day-old etiolated** ***ros1-4*** **mutant combinations.**
(PPTX)

Table S5 **List of Arabidopsis mutants and primers used in the study.**
(DOC)

## References

[1] Law JA, Jacobsen SE (2010) Establishing, maintaining and modifying DNA methylation patterns in plants and animals. Nat Rev Genet 11: 204–220. 20142834 10.1038/nrg2719 PMC3034103

[2] Walker EL (1998) Paramutation of the r1 locus of maize is associated with increased cytosine methylation. Genetics 148: 1973–1981. 9560410 10.1093/genetics/148.4.1973 PMC1460097

[3] Walker EL, Panavas T (2001) Structural features and methylation patterns associated with paramutation at the r1 locus of Zea mays. Genetics 159: 1201–1215. 11729163 10.1093/genetics/159.3.1201 PMC1461878

[4] Haring M, Bader R, Louwers M, Schwabe A, van Driel R, et al (2010) The role of DNA methylation, nucleosome occupancy and histone modifications in paramutation. Plant J 63: 366–378. 20444233 10.1111/j.1365-313X.2010.04245.x

[5] Chandler VL, Stam M (2004) Chromatin conversations: Mechanisms and implications of paramutation. Nat Rev Genet 5: 532–544. 15211355 10.1038/nrg1378

[6] Arteaga-Vazquez MA, Chandler VL (2010) Paramutation in maize: RNA mediated trans-generational gene silencing. Curr Opin Genet Dev 20: 156–163. 20153628 10.1016/j.gde.2010.01.008 PMC2859986

[7] Coe EH (1966) Properties Origin and Mechanism of Conversion-Type Inheritance at B Locus in Maize. Genetics 53: 1035–&. 10.1093/genetics/53.6.1035 PMC1211079 17248307

[8] Patterson GI, Kubo KM, Shroyer T, Chandler VL (1995) Sequences Required for Paramutation of the Maize B-Gene Map to a Region Containing the Promoter and Upstream Sequences. Genetics 140: 1389–1406. 7498778 10.1093/genetics/140.4.1389 PMC1206702

[9] Stam M, Belele C, Dorweiler JE, Chandler VL (2002) Differential chromatin structure within a tandem array 100 kb upstream of the maize b1 locus is associated with paramutation. Genes Dev 16: 1906–1918. 12154122 10.1101/gad.1006702 PMC186425

[10] Rassoulzadegan M, Grandjean V, Gounon P, Vincent S, Gillot I, et al (2006) RNA-mediated non-mendelian inheritance of an epigenetic change in the mouse. Nature 441: 469–474. 16724059 10.1038/nature04674

[11] Alleman M, Sidorenko L, McGinnis K, Seshadri V, Dorweiler JE, et al (2006) An RNA-dependent RNA polymerase is required for paramutation in maize. Nature 442: 295–298. 16855589 10.1038/nature04884

[12] Erhard KF Jr, Stonaker JL, Parkinson SE, Lim JP, Hale CJ, et al (2009) RNA polymerase IV functions in paramutation in Zea mays. Science 323: 1201–1205. 19251626 10.1126/science.1164508

[13] Sidorenko L, Dorweiler JE, Cigan AM, Arteaga-Vazquez M, Vyas M, et al (2009) A Dominant Mutation in mediator of paramutation2, One of Three Second-Largest Subunits of a Plant-Specific RNA Polymerase, Disrupts Multiple siRNA Silencing Processes. PLoS Genetics 5: e1000725. 19936058 10.1371/journal.pgen.1000725 PMC2774164

[14] Erhard KF, Hollick JB (2011) Paramutation: a process for acquiring trans-generational regulatory states. Curr Opin Plant Biol 14: 210–216. 21420347 10.1016/j.pbi.2011.02.005

[15] Lindroth AM, Cao X, Jackson JP, Zilberman D, McCallum CM, et al (2001) Requirement of CHROMOMETHYLASE3 for maintenance of CpXpG methylation. Science 292: 2077–2080. 11349138 10.1126/science.1059745

[16] Cao X, Jacobsen SE (2002) Role of the arabidopsis DRM methyltransferases in de novo DNA methylation and gene silencing. Curr Biol 12: 1138–1144. 12121623 10.1016/s0960-9822(02)00925-9

[17] Chan SWL, Henderson IR, Jacobsen SE (2005) Gardening the genome: DNA methylation in Arabidopsis thaliana. Nat Rev Genet 6: 351–360. 15861207 10.1038/nrg1601

[18] Gong ZH, Morales-Ruiz T, Ariza RR, Roldan-Arjona T, David L, et al (2002) ROS1, a repressor of transcriptional gene silencing in Arabidopsis, encodes a DNA glycosylase/lyase. Cell 111: 803–814. 12526807 10.1016/s0092-8674(02)01133-9

[19] Penterman J, Uzawa R, Fischer RL (2007) Genetic interactions between DNA demethylation and methylation in Arabidopsis. Plant Physiol 145: 1549–1557. 17951456 10.1104/pp.107.107730 PMC2151691

[20] Matzke MA, Primig M, Trnovsky J, Matzke AJM (1989) Reversible Methylation and Inactivation of Marker Genes in Sequentially Transformed Tobacco Plants. Embo J 8: 643–649. 16453872 10.1002/j.1460-2075.1989.tb03421.x PMC400855

[21] Daxinger L, Hunter B, Sheik M, Jauvion V, Gasciolli V, et al (2008) Unexpected silencing effects from T-DNA tags in Arabidopsis. Trends Plant Sci 13: 4–6. 18178509 10.1016/j.tplants.2007.10.007

[22] Stuart JM, Segal E, Koller D, Kim SK (2003) A gene-coexpression network for global discovery of conserved genetic modules. Science 302: 249–255. 12934013 10.1126/science.1087447

[23] Mutwil M, Usadel B, Schutte M, Loraine A, Ebenhoh O, et al (2010) Assembly of an Interactive Correlation Network for the Arabidopsis Genome Using a Novel Heuristic Clustering Algorithm. Plant Phys 152: 29–43. 10.1104/pp.109.145318 PMC2799344 19889879

[24] Ko JH, Kim JH, Jayanty SS, Howe GA, Han KH (2006) Loss of function of COBRA, a determinant of oriented cell expansion, invokes cellular defence responses in Arabidopsis thaliana. J Exp Bot 57: 2923–2936. 16873454 10.1093/jxb/erl052

[25] Fagard M, Desnos T, Desprez T, Goubet F, Refregier G, et al (2000) PROCUSTE1 encodes a cellulose synthase required for normal cell elongation specifically in roots and dark-grown hypocotyls of arabidopsis. Plant Cell 12: 2409–2423. 11148287 10.1105/tpc.12.12.2409 PMC102227

[26] Cano-Delgado A, Penfield S, Smith C, Catley M, Bevan M (2003) Reduced cellulose synthesis invokes lignification and defense responses in Arabidopsis thaliana. Plant J 34: 351–362. 12713541 10.1046/j.1365-313x.2003.01729.x

[27] Schindelman G, Morikami A, Jung J, Baskin TI, Carpita NC, et al (2001) COBRA encodes a putative GPI-anchored protein, which is polarly localized and necessary for oriented cell expansion in Arabidopsis. Genes Dev 15: 1115–1127. 11331607 10.1101/gad.879101 PMC312689

[28] Roudier F, Fernandez AG, Fujita M, Himmelspach R, Borner GH, et al (2005) COBRA, an Arabidopsis extracellular glycosyl-phosphatidyl inositol-anchored protein, specifically controls highly anisotropic expansion through its involvement in cellulose microfibril orientation. Plant Cell 17: 1749–1763. 15849274 10.1105/tpc.105.031732 PMC1143074

[29] Bird A (2002) DNA methylation patterns and epigenetic memory. Genes Dev 16: 6–21. 11782440 10.1101/gad.947102

[30] Henderson IR, Jacobsen SE (2007) Epigenetic inheritance in plants. Nature 447: 418–424. 17522675 10.1038/nature05917

[31] Santi DV, Garrett CE, Barr PJ (1983) On the Mechanism of Inhibition of DNA Cytosine Methyltransferases by Cytosine Analogs. Cell 33: 9–10. 6205762 10.1016/0092-8674(83)90327-6

[32] Baubec T, Pecinka A, Rozhon W, Mittelsten Scheid O (2009) Effective, homogeneous and transient interference with cytosine methylation in plant genomic DNA by zebularine. Plant J 57: 542–554. 18826433 10.1111/j.1365-313X.2008.03699.x PMC2667684

[33] Yoshida M, Kijima M, Akita M, Beppu T (1990) Potent and Specific-Inhibition of Mammalian Histone Deacetylase Both Invivo and Invitro by Trichostatin-A. J Biol Chem 265: 17174–17179. 2211619

[34] Kankel MW, Ramsey DE, Stokes TL, Flowers SK, Haag JR, et al (2003) Arabidopsis MET1 cytosine methyltransferase mutants. Genetics 163: 1109–1122. 12663548 10.1093/genetics/163.3.1109 PMC1462485

[35] Saze H, Mittelsten Scheid O, Paszkowski J (2003) Maintenance of CpG methylation is essential for epigenetic inheritance during plant gametogenesis. Nat Genet 34: 65–69. 12669067 10.1038/ng1138

[36] Penterman J, Zilberman D, Huh JH, Ballinger T, Henikoff S, et al (2007) DNA demethylation in the Arabidopsis genome. Proc Natl Acad Sci U S A 104: 6752–6757. 17409185 10.1073/pnas.0701861104 PMC1847597

[37] Lister R, O’Malley RC, Tonti-Filippini J, Gregory BD, Berry CC, et al (2008) Highly integrated single-base resolution maps of the epigenome in Arabidopsis. Cell 133: 523–536. 18423832 10.1016/j.cell.2008.03.029 PMC2723732

[38] Gao Y, Zhao Y (2012) Epigenetic suppression of T-DNA insertion mutants in Arabidopsis. Mol Plant, advance access *doi: 10.1093/mp/sss093*. 10.1093/mp/sss093 PMC3716301 22973063

[39] Nakagawa T, Kurose T, Hino T, Tanaka K, Kawamukai M, et al (2007) Development of series of gateway binary vectors, pGWBs, for realizing efficient construction of fusion genes for plant transformation. J Biosci Bioeng 104: 34–41. 17697981 10.1263/jbb.104.34

[40] Livak KJ, Schmittgen TD (2001) Analysis of relative gene expression data using real-time quantitative PCR and the 2(T)(-Delta Delta C) method. Methods 25: 402–408. 11846609 10.1006/meth.2001.1262

[41] Urdvardi MK, Czechowski T, Scheible WR (2008) Eleven golden rules of quantitative RT-PCR. Plant Cell 20: 1736–1737. 18664613 10.1105/tpc.108.061143 PMC2518243

[42] Hong SM, Bahn SC, Lyu A, Jung HS, Ahn JH (2010) Identification and testing of superior reference genes for a starting pool of transcript normalisation in Arabidopsis. Plant Cell Physiol. 51: 1964–1706. 10.1093/pcp/pcq128 20798276

[43] Updegraff DM (1960) Semimicro determination of cellulose in biological materials. Anal Biochem 32: 420–424. 10.1016/s0003-2697(69)80009-6 5361396

[44] Dische Z (1962) Colour reactions of carbohydrates. Methods in Carbohydrate Chemistry, Whistler R.L., Wolfrom M.L. (Eds), Vol. 1. Academic Press Inc, New York, NY, 478–548.

