# Supplementary figures and images for: Paramutation-Like Interaction of T-DNA Loci in Arabidopsis
Source: PLoS One. 2012 Dec 14;7(12):e51651. doi: 10.1371/journal.pone.0051651 (PMC3522736; doi:10.1371/journal.pone.0051651)

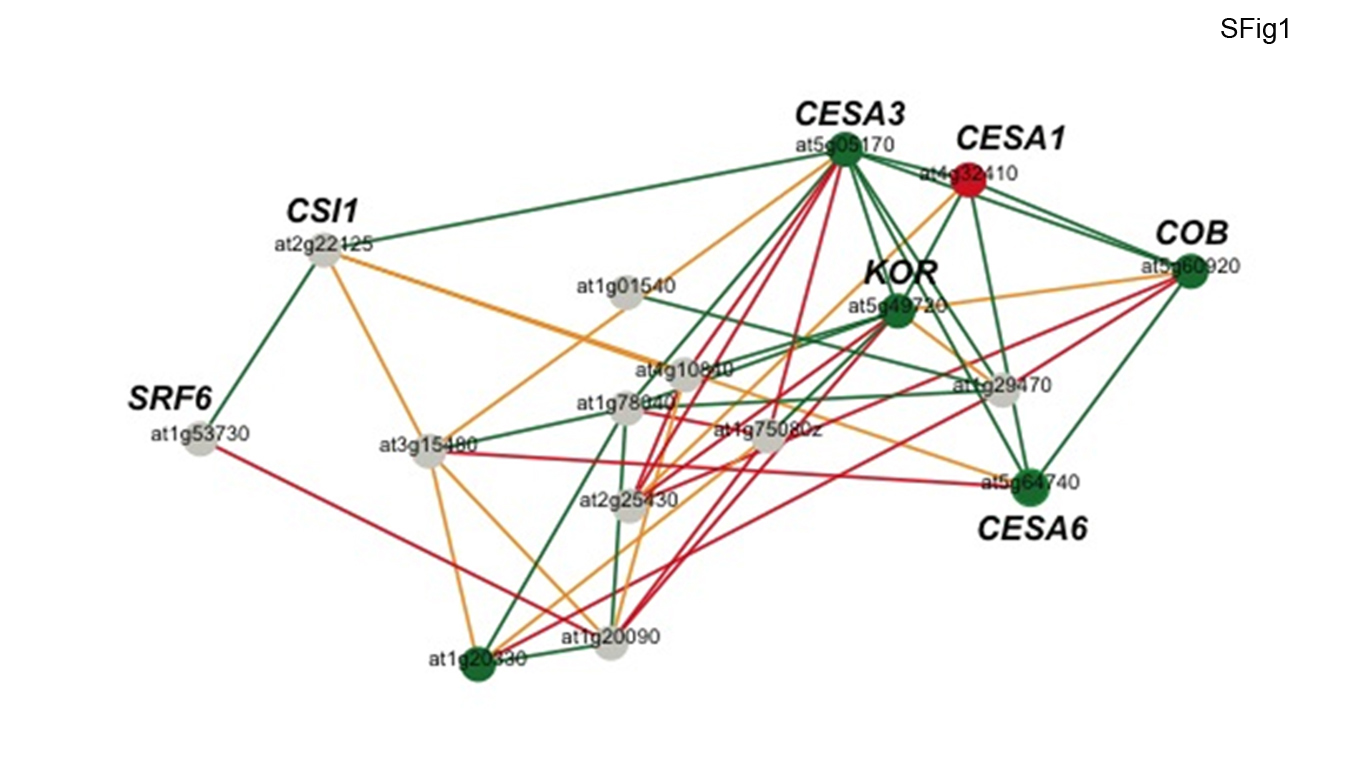

Supplement: Figure S1 — Truncated co-expression network from Cluster 86 in [23] . Brief annotations of genes are indicated in black text. Different coloured edges indicate strength of transcriptional coordination. Green; mutual rank ≤10, Orange; mutual rank ≤20, Red; mutual rank ≤30. Low mutual rank indicates stronger co-expression relationships. Coloured nodes indicate embryo lethality (red), other described phenotypes (green), and no reported phenotype (grey) of mutants corresponding to the respective gene. (TIF) [file pone.0051651.s001.tif]

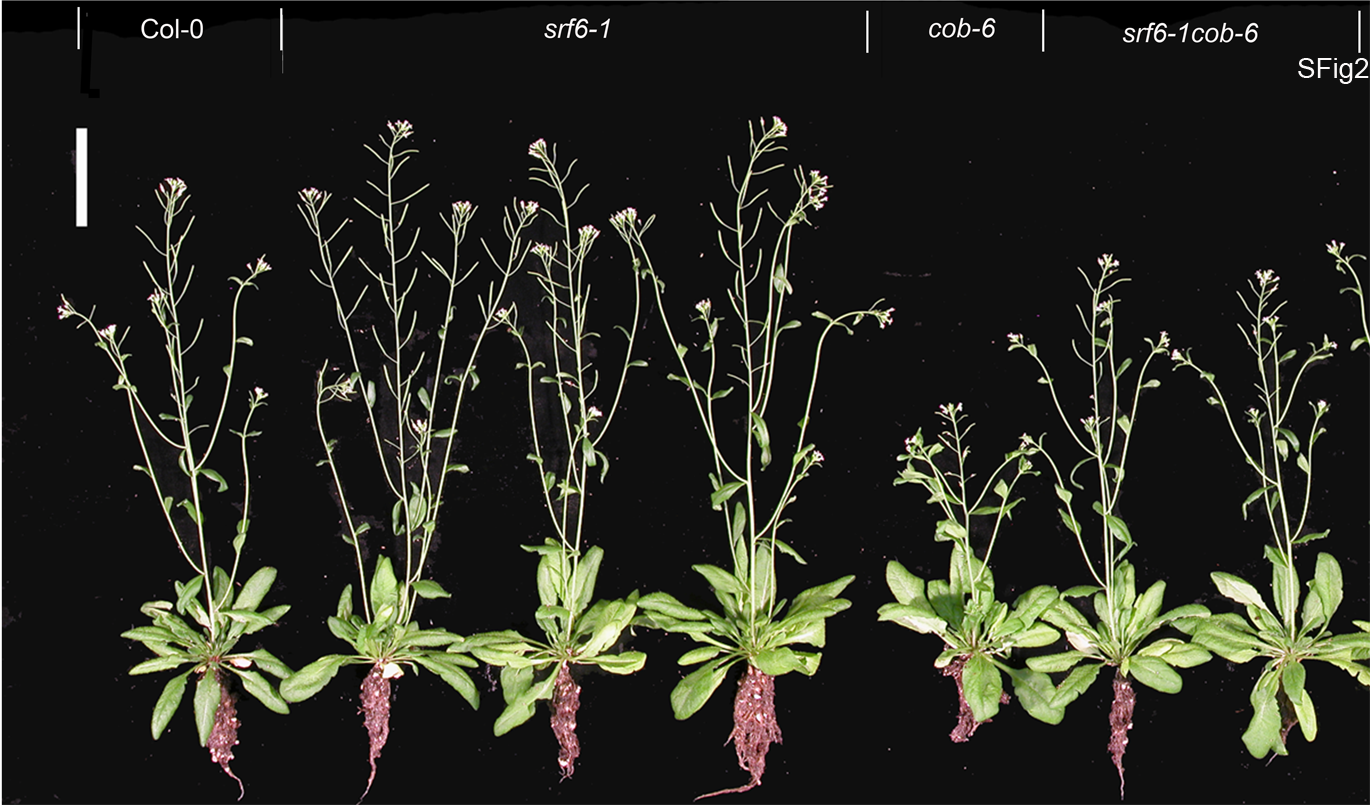

Supplement: Figure S2 — Phenotype of six-week-old Col-0, srf6-1, cob-6 and srf6-1cob-6 plants grown in 16-h light, 8-h dark. Scale bar 5 cm. (TIF) [file pone.0051651.s002.tif]

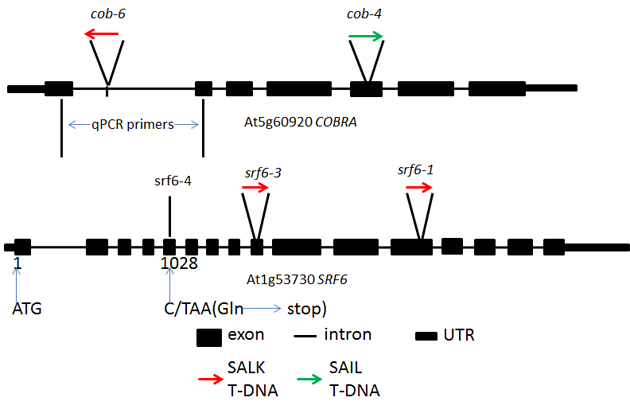

Supplement: Figure S3 — Location of the premature stop codon in srf6-4 and the T-DNA insertions in COBRA and SRF6 . (TIFF) [file pone.0051651.s003.tiff]

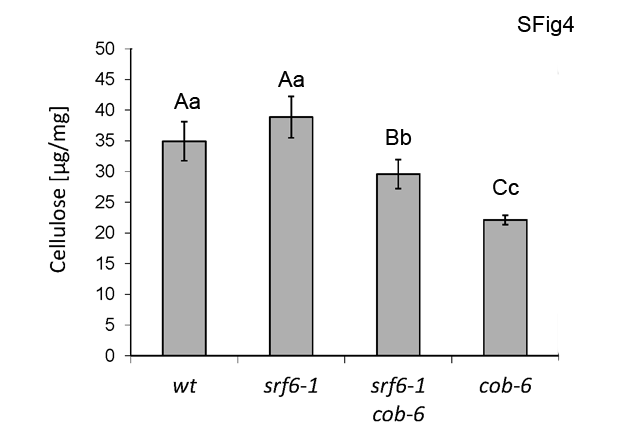

Supplement: Figure S4 — Cellulose content in four-day-old dark grown seedlings. Genotypes, mean and SE are indicated. A, B, and C indicate significant difference of the genotypes ranked by Duncan’s test at P<0.01, a, b, c indicate ranking by Duncan test at P≤0.05. (TIFF) [file pone.0051651.s004.tiff]

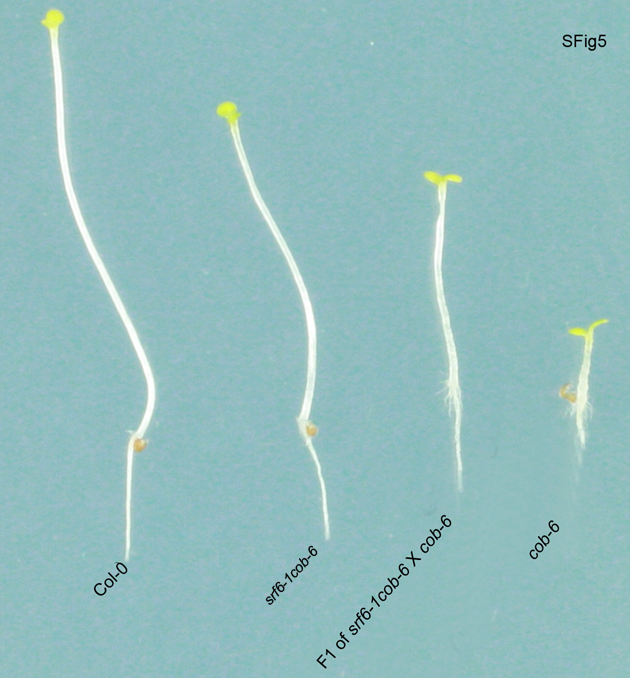

Supplement: Figure S5 — The phenotype of etiolated F1 seedlings derived from the cross between srf6-1cob-6 and cob-6 . Picture is representative of multiple seedlings. (TIF) [file pone.0051651.s005.tif]

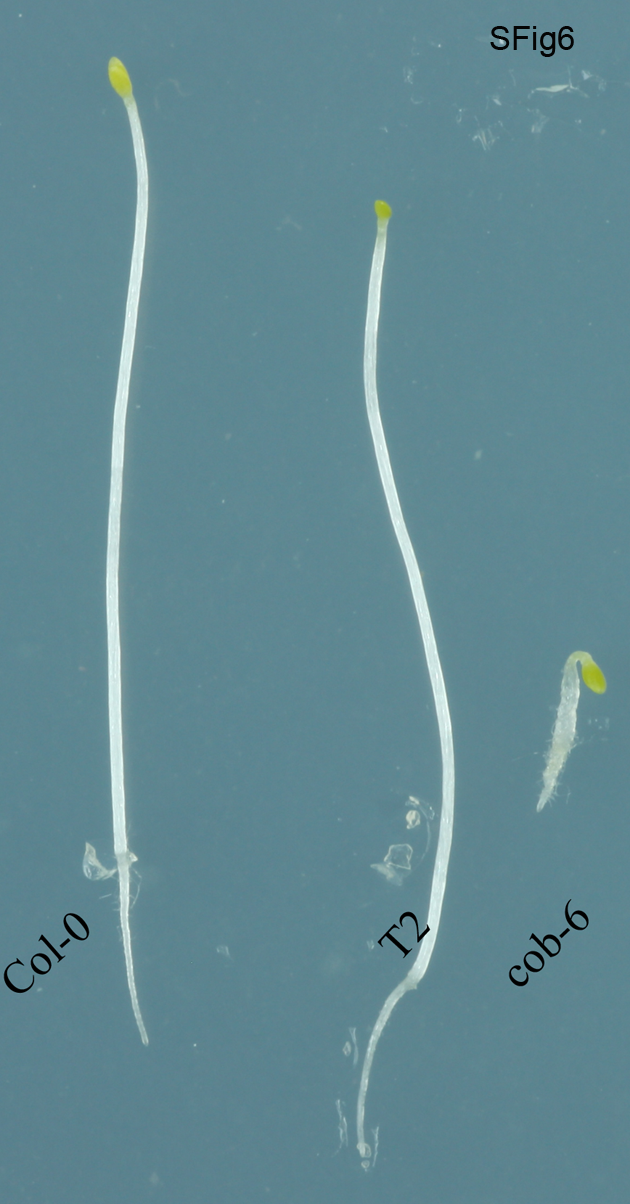

Supplement: Figure S6 — Complementation of cob-6 with a genomic COBRA construct. (TIF) [file pone.0051651.s006.tif]

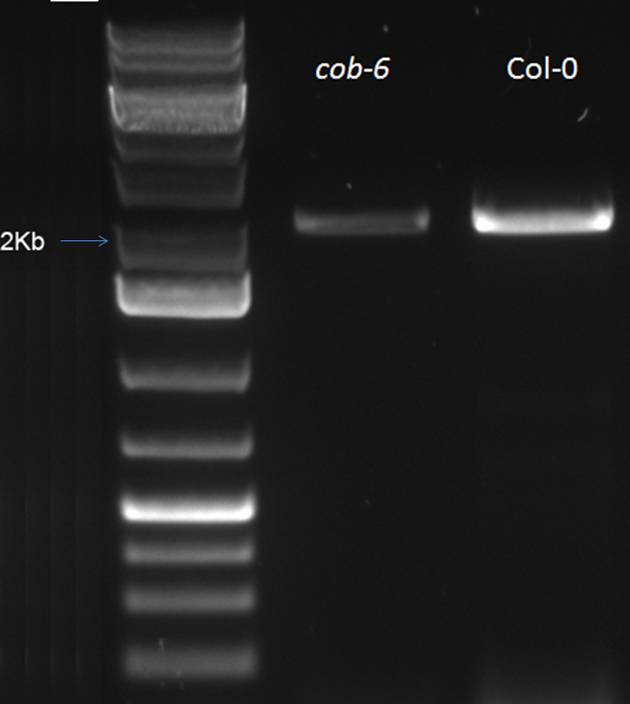

Supplement: Figure S7 — Amplification of full length COBRA cDNA from cob-6 and Col-0. (TIF) [file pone.0051651.s007.tif]

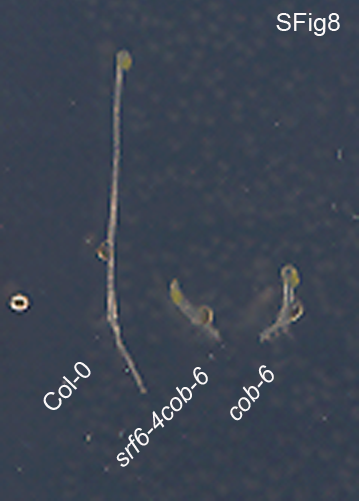

Supplement: Figure S8 — Phenotype comparison of the srf6-4cob-6 and cob-6 etiolated seedlings. Picture is representative of multiple seedlings. (TIF) [file pone.0051651.s008.tif]

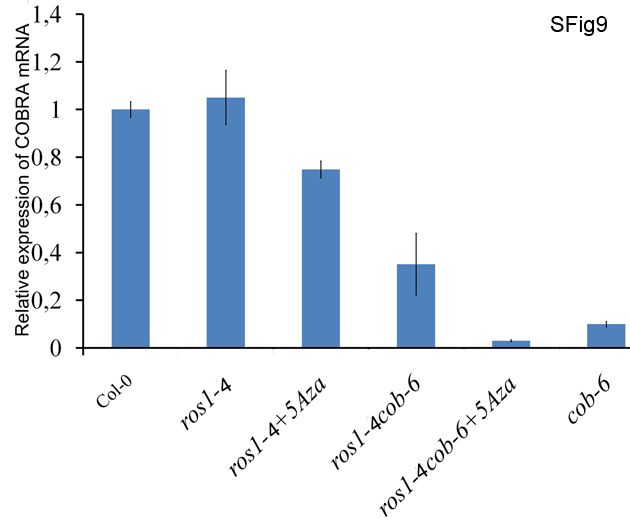

Supplement: Figure S9 — The effect of DNA demethylase ros1-4 mutation on COBRA transcript levels. Also shown is the effect of DNA methylation inhibitor 5-azacytidine (5-Aza) on COBRA expression in ros1-4 and ros1-4cob-6. Genotypes, mean and SE are indicated. RNA was extracted from etiolated seedlings, n = 3 pools of seedlings. (TIF) [file pone.0051651.s009.tif]
