# Supplementary material for: Paramutation-Like Interaction of T-DNA Loci in Arabidopsis
Source: PLoS One. 2012 Dec 14;7(12):e51651. doi: 10.1371/journal.pone.0051651 (PMC3522736; doi:10.1371/journal.pone.0051651)
Supplement: Table S5 — List of Arabidopsis mutants and primers used in the study. (DOC) [file pone.0051651.s014.doc]

**Table S5.** List of the *Arabidopsis thaliana* mutants and primers.

**Mutant lines used in the study**

| **Name** | **Locus** | **Mutation** |
| --- | --- | --- |
| *eli1-1*  *prc1-1* | AT5G05170  AT5G64740 | EMS line (C to T)  EMS line (C to T) |
| *srf6-1* | AT1G53730 | SALK_054337c |
| *srf6-3* | AT1G53730 | SALK_077702 |
| *srf6-4* | AT1G53730 | EMS TILLING line (C to T) |
| *cob-6* | AT5G60920 | SALK_051906 |
| *cob-4* | AT5G60920 | SAIL_735D10 |
| *drm1-2* | AT5G15380 | SALK_031705 |
| *drm2-2* | AT5G14620 | SALK_150863 |
| *cmt3-11* | AT1G69770 | SALK_148381 |
| *met1-3* | AT5G49160 | Provided by Ian Henderson , University of Cambridge, UK |
| *ddm1* | AT5G66750 | SALK_000590 |
| *srf4-1* | AT3G13065 | SAIL_253_A09 |
| *ros1-4* | [AT2G36490](http://www.arabidopsis.org/servlets/TairObject?id=32321&type=locus) | SAIL_186_C01 |
| *r1* | AT2G30980 | SALK_072480* |
| *r2* | AT1G56340 | SALK_055452* |
| *r3* | AT2G35050 | SALK_107170* |
|  |  |  |

*Lines used to test for the *trans* SALK t-dna effect.

**Primers used in the study**

| **Primer name** | **Sequence** | **Use for** |
| --- | --- | --- |
| cob-6 LP | TTTGTGCTCCAACCATACTCC | srf6 T-DNA mutant screening |
| cob-6 RP | TGTGTGCATACCGTGAAAGTC | srf6 T-DNA mutant screening |
| srf6-3LP | TCGAGTTTATAACCGTCGGTG | srf6 T-DNA mutant screening |
| srf6-3RP | TGTGTGCATACCGTGAAAGTC | srf6 T-DNA mutant screening |
| cob6-LP | TTTGTGCTCCAACCATACTCC | cob-6 T-DNA mutant screening |
| cob6-RP | AAGCAAAGCACCTTCCTCTTC | cob-6 T-DNA mutant screening |
| cob4_LP | TTTGTTTGTTCGATCTAGGCC | cob4 T-DNA mutant screening |
| cob4-RP | TTTACATGCCAATGCACTCTG | cob4 T-DNA mutant screening |
| drm1-2 RP | GTCGATGGAGTGCAACTTCTC | drm1-2 T-DNA mutant screening |
| drm1-2LP | CCTGTGTTGATTGGGATTCAG | drm1-2 T-DNA mutant screening |
| drm2-2 RP | TTGTCGCAAAAAGCAAAAGAG | drm2-2 T-DNA mutant screening |
| drm2-2 LP | AGATCGCTTCCAGAGTTAGCC | drm2-2 T-DNA mutant screening |
| cmt3-11RP | ATAAGAGAAGGAGCTGCTGCC | cmt3-11 T-DNA mutant screening |
| cmt3-11LP  prc1-1LP  prc1-1RP | CCCTCAACAATTAACTGACGC  GACAGTGGCTGCGGATAAGAAG  GAATATCTTCGGTAACAGAACC | cmt3-11 T-DNA mutant screening  prc1-1 mutant screening  prc1-1 mutant screening |
| IRH_1 | GATTGTGTCTCTACTACAGAGGC | met1-3 T-DNA mutant screening |
| IRH_2 | GTTAAGCTCATTCATAGCCTTGC | met1-3 T-DNA mutant screening |
| IRH_3 | TGGACGTGAATGTAGACACGTCG | met1-3 T-DNA mutant screening |
| ros1-4LP | CGTGGATTACATAACATGTTATTTG | ros1-3 T-DNA mutant screening |
| ros1-4RP | TTGTTCCCAACAAATCTCCTG | ros1-3 T-DNA mutant screening |
| LBb1.3 | ATTTTGCCGATTTCGGAAC | SALK T-DNA left border |
| LB_GabiKat | ATAATAACGCTGCGGACATCTACATTT | GabiKat T-DNA left border |
| COB1QPCRfwd | TTTCTTGGCCTTATGGATCG | cobra QPCR |
| COBQPCRrev | CCATCAGGAGTCCAGCTCAT | cobra QPCR |
| Helicase-F | CCATTCTACTTTTTGGCGGCT | reference gene QPCR |

| Helicase-R | TCAATGGTAACTGATCCACTCTGATG | reference gene QPCR |
| --- | --- | --- |
| attB1 cobgenomic | GGGGACAAGTTTGTACAAAAAAGCAGGCTTATTAAGATTTGACTGATTT | cobra complementation |
| attB2 cobgenomic | GGGGACCACTTTGTACAAGAAAGCTGGGTATTTAGATCACAGTAAGTC | cobra complementation |
|  |  |  |
